# Genomic analyses reveal presence of extensively drug-resistant *Salmonella enterica* serovars isolated from clinical samples in Guizhou province, China, 2019–2023

**DOI:** 10.3389/fmicb.2025.1532036

**Published:** 2025-03-27

**Authors:** Yongxian Wen, Jingtong Wu, Lv You, Xiaoyu Wei, Junhua Wang, Shijun Li

**Affiliations:** ^1^School of Public Health, The Key Laboratory of Environmental Pollution Monitoring and Disease Control, Ministry of Education, Guizhou Medical University, Guiyang, China; ^2^Laboratory of Bacterial Disease, Experimental Center, Guizhou Provincial Center for Disease Control and Prevention, Guiyang, China

**Keywords:** *Salmonella*, extensively drug-resistant, plasmid, antimicrobial resistance genes, whole-genome sequencing

## Abstract

**Background:**

The emergence of extensively drug-resistant (XDR) *Salmonella* in humans poses a significant public health and therapeutic challenge. However, limited data are available on XDR *Salmonella* isolates from Guizhou province, China. This study aimed to investigate the molecular epidemiology and resistance patterns of XDR *Salmonella* isolates from clinical samples in this region.

**Methods:**

A total of 931 *Salmonella* isolates were screened for XDR isolates through antimicrobial susceptibility testing. These XDR isolates were subjected to whole-genome sequencing (WGS) and bioinformatic analysis to further systematically investigating the molecular epidemiology and resistance patterns of XDR *Salmonella* isolates.

**Results:**

Between 2019 and 2023, 931 *Salmonella* isolates were collected from clinical samples in Guizhou. Of these isolates, 51 (5.5%) were identified as XDR and classified into 16 serovars. Among the serovars, 15 corresponded to a specific sequence type, except for *S. Typhimurium* serovars. The predominant serovars, *S*. 1,4,[5],12:i:-, *S. Enteritidis*, and *S*. Kentucky, were divided into ST34, ST11, and ST198, respectively. Genomic analysis showed that all XDR isolates harbored at least eight antimicrobial resistance genes (ARGs) and multidrug efflux pumps. Highly prevalent point mutations in *gyrA* (D87 and S83) and *parC* (S80I) were detected, along with eight plasmid-mediated quinolone resistance (*PMQR*) genes. The *qnrS1* gene was the most common (43.1%), followed by *oqxA*, *aac-(6′)-lb-cr* variant, *qnrB4*, *qnrS2*, *qnrA1*, *qepA2*, and *oqxB*. The predominant *β*-lactamase gene was *bla_TEM-1_* (54.9%), and *bla_CTX-M-55_* (35.3%) was the most prevalent extended-spectrum *β*-lactamase subtype. Notably, *bla_NDM-1_* gene was identified for the first time in *Salmonella* from Guizhou, and one *S.* 1,4,[5],12:i:- isolate contained the *mcr-1.1* gene. ARGs profiles varied by serovars, with *S.* 1,4,[5],12:i:- isolates carrying the highest number. Ten plasmid types were identified, predominantly IncHI2/IncHI2A (47.5%). Key resistance genes such as *tetA*, *PMQR*, *bla_CTX-M_*, *mcr-1.1*, and *bla_NDM-1_* were located on IncHI2/IncHI2A plasmids. Notably, 75.0% of the conjugative plasmids belonged to IncHI2/IncHI2A, indicating that horizontal gene transfer through conjugation facilitates ARGs dissemination. Core genome multilocus sequence typing (cgMLST) analysis revealed significant genetic diversity, with 39 core genome sequence types (cgSTs) identified and no evidence of outbreaks.

**Conclusion:**

The rising prevalence of XDR *Salmonella* in Guizhou province is concerning. Initial whole-genome sequencing (WGS) data provide critical insights for understanding and controlling XDR *Salmonella* infections, aiding public health officials in identifying emerging threats and trends.

## 1 Introduction

Non-typhoidal *Salmonella* (NTS) has emerged as a leading cause of infectious diarrhea globally, underscoring its significance as both a foodborne and zoonotic pathogen. Reports indicated an alarming prevalence of NTS invasive disease, with an estimated 535,000 cases and 77,500 deaths attributed to this pathogen in 2017 alone, which indicated a mean all-age case fatality rate of approximately 14.5% (GBD 2017 Non-Typhoidal Salmonella Invasive Disease Collaborators, 2019). In the United States, the annual incidence was staggering, with around 1.35 million cases of NTS reported, resulting in approximately 26,500 hospitalizations and 420 fatalities (Lewis et al., 2023). In recent years, certain high-income countries like the United States, Australia, and Sweden have reported outbreaks of foodborne diseases (Ford et al., 2023; Kerr et al., 2022; Jansson Mörk et al., 2022). Additionally, Europe ranked NTS as the second most common zoonosis (European Food Safety Authority (EFSA) and European Centre for Disease Prevention and Control (ECDC), 2023), while data from the Chinese Center for Disease Control and Prevention (CDC) revealed that up to 80% of bacterial foodborne illnesses stem from *Salmonella*, highlighting its pervasive role in public health crises across various regions (Hu et al., 2022; Wang et al., 2021). In Guizhou province, food poisoning incidents attributable to *Salmonella* are common, underscoring the pathogen’s status as the leading cause of foodborne illness and infectious diarrhea in the region (Wei et al., 2023; Zhou et al., 2024). Studies have identified *Salmonella* as a prevalent etiological agent in diarrheal diseases across Guizhou province, revealing a significant public health threat that necessitates urgent attention and intervention.

The clinical manifestations of salmonellosis generally include diarrhea, fever, abdominal cramps, and vomiting, illustrating that while the disease is often self-limiting, certain populations—particularly those with compromised immune systems—are susceptible to severe complications (Wang Z. et al., 2024). While antimicrobial treatment was frequently unnecessary in most cases, its importance escalates in severe infections, especially amongvulnerable populations. Alarmingly, the global rise of antimicrobial resistance (AMR) poses a significant threat, with XDR *Salmonella* isolates emerging across multiple countries, including China, Australia, Italy, and Egypt (Chen et al., 2022; Liu B. et al., 2024; Cummins et al., 2020; Russo et al., 2022; Algammal et al., 2023; Sallam et al., 2024). The prevalence of XDR *Salmonella*, drastically limited treatment options and exacerbated clinical outcomes, emphasizing an urgent need for comprehensive understanding of the molecular mechanisms that underpin antibiotic resistance. In Guizhou province, China, the situation is particularly critical, as evidenced by a multidrug resistance rate of 86.7% among clinical *Salmonella* isolates from 2013 to 2017, with 4.4% categorized as extensively drug-resistant (Wei et al., 2023). Recent study on food revealed that a significant proportion of *Salmonella* isolates from retail food sources exhibited resistance to multiple antibiotic classes (Zhou et al., 2024). These data highlights the importance of understanding the underlying resistance mechanisms that facilitate the survival and proliferation of *Salmonella*. However, the available literature was lacking in comprehensive analyses of the molecular epidemiological characteristics and prevalence of resistance genes among clinical XDR *Salmonella* isolates in Guizhou. The gap in information limited the development of effective intervention strategies.

This study aimed to fill this critical gap by systematically investigating the molecular epidemiology and resistance patterns of XDR *Salmonella* isolates from clinical samples in Guizhou province. By exploring the incidence rates of resistance genes in clinical isolates, the findings served as a pivotal reference for public health strategies to prevent and manage *Salmonella* infections. Furthermore, this research informed the judicious use of antimicrobial therapies, contributing to efforts to mitigate the burgeoning public health challenge of antimicrobial resistance.

## 2 Materials and methods

### 2.1 XDR *Salmonella* isolate, serotyping and antimicrobial susceptibility testing

A surveillance program specifically targeting non-typhoidal *Salmonella* was implemented based on laboratory analyses. A total of 931 *Salmonella* isolates were identified from stool samples collected from diarrhea patients in 109 hospitals across six cities and three prefectures of Guizhou province between January 1, 2019 and December 31, 2023, as described previously (Wu et al., 2025). These isolates were distributed in Guiyang (*n* = 164), Zunyi (*n* = 148), Liupanshui (*n* = 90), Anshun (*n* = 45), Bijie (*n* = 33), Tongren (*n* = 258), Qiandongnan (*n* = 72), Qiannan (*n* = 89), and Qianxinan (*n* = 32). Additionally, the *Salmonella* isolates were dispatched to the bacterial laboratory of the Guizhou Provincial Center for Disease Control and Prevention for further identification and analysis. The serovars of *Salmonella* isolates were determined using the slide agglutination method according to the Kauffmann-White-Le Minor scheme (Grimont and Weill, 2007; Guibourdenche et al., 2010). Antimicrobial susceptibility testing was conducted and interpreted using the broth microdilution method, following the Clinical & Laboratory Standards Institute guidelines (CLSI, 2023) (Supplementary Data S1). The following antimicrobials were tested using customized antimicrobial susceptibility test plate CHNENF (Thermo Fisher Scientific, America): (i) chloramphenicol (C), (ii) sulfonamides: trimethoprim-sulfamethoxazole (SXT), (iii) quinolones: nalidixic acid (NA), ciprofloxacin (CIP), (iv) macrolides: azithromycin (AZM), (v) aminoglycosides: streptomycin (STS), amikacin (AMK), (vi) tetracyclines: tetracycline (TE), tigecycline (TGC), (vii) cephems: cefotaxime (CTX), ceftazidime (CAZ), (vii) *β*-lactamase inhibitor: ceftazidime/avibactam (CZA), ampicillin/sulbactam (SAM), (ix) carbapenems: ertapenem (ETP), meropenem (MEM), (x) penicillin: ampicillin (AM), (xi) lipopeptide: colistin (CL). *Escherichia coli* ATCC 25922 was used for quality control. Sterile saline solution was used as the negative control group. XDR *Salmonella* was defined as non-susceptibility to at least one agent in all but two or fewer antimicrobial categories (Magiorakos et al., 2012). Antimicrobial resistance, including antimicrobial resistance rates and resistance profiles of XDR *Salmonella* isolates, was analyzed using Whonet 2023 software.^1^

### 2.2 DNA extraction and whole genome sequencing

Genomic DNA extraction was performed for XDR *Salmonella* isolates using the magnetic bacteria genomic DNA kit (Baiju Technologies, China). The extracted DNA was precisely quantified with the Qubit 4 Fluorometer (Thermo-Scientific, Waltham, United States) in accordance with the manufacturer’s instructions. High-quality genomic DNA were further conducted for the subsequent ligation step. Adapter ligation and clean-up of sequences were performed using the SQK-NBD114.24 kit. After that, sequencing was carried out on the long-read Oxford Nanopore GridION platform (Nanopore Technologies, United Kingdom). High-coverage data were generated for downstream analyses. Species identification was performed using Kraken 2 V2.1.2,^2^ whole genome assembly was conducted with Flye V2.8.3,^3^ and the assembly was polished using medaka V1.6.0,^4^ which resulted in the generation of a complete genome sequence.

### 2.3 MLST analysis, serovars prediction, ARGs identification, and plasmid characterization

The serovars and multi-locus sequence typing (MLST) of XDR *Salmonella* isolates were predicted using SeqSero 2 V1.1.0^5^ and MLST V2.0.^6^ The complete genomic sequences of XDR *Salmonella* isolates were aligned with the Comprehensive Antibiotic Resistance Gene Database (CARD)^7^ to obtain the resistance genes. Plasmids were predicted using PlasFlow V1.1,^8^ and the replicon family of the predicted plasmids was determined using PlasmidFinder V2.1.^9^ To precisely know the location of ARGs, we submitted the identified plasmid sequences and chromosomal sequences separately to the CARD for obtaining the corresponding ARGs. The transferability was predicted using VR profile 2 (Wang et al., 2022). Batch analysis of XDR *Salmonella* plasmids was conducted using oriTfinder to ascertain the presence or absence of oriTs, relaxase genes, T4CP genes, and T4SS gene clusters (Li et al., 2018). Moreover, the oriT, relaxase, and T4CP genes type were identified by referring to the oriTDB database.^10^ In addition, T4SS genes cluster types were classified according to the SecReT4 database.^11^

### 2.4 Core genome multilocus sequence typing (cgMLST) analysis

The gene sequences of XDR *Salmonella* isolates from Guizhou province were screened and analyzed using cgMLST finder V1.2.0^12^ to assess the core genome multi-locus sequence typing (cgMLST) locus dataset. A Maximum Likelihood (ML) tree was constructed with IQtree V2.3.5^13^ and visualized using the TVBOT online website^14^ (Xie et al., 2023) to create a binary image for comparative analysis of resistance.

## 3 Result

### 3.1 Antimicrobial resistance

**Figure 1:**
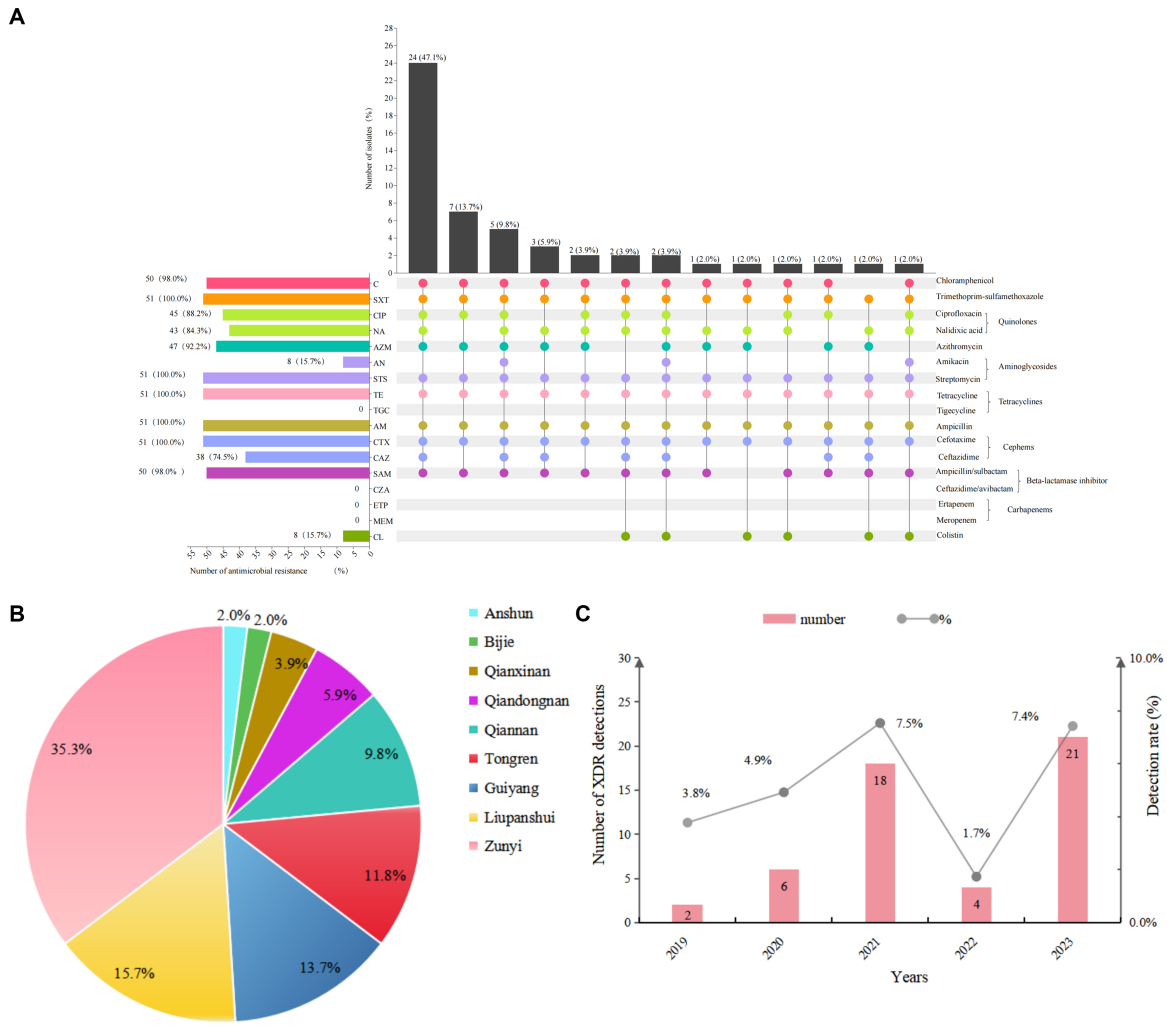
Antimicrobial resistance (AMR) phenotypes of 51 XDR *Salmonella* isolates. **(A)** The antibiotic resistance profiles of XDR *Salmonella* isolates were visualized using an UpSet plot. In the combination matrix positioned beneath the primary bar chart, individual columns corresponded to distinct AMR phenotypic profiles. Each colored dot within these columns represented resistance to a specific antimicrobial agent, with unique color coding different antimicrobial. The vertical bar chart displayed the total number of isolates exhibiting particular resistance combinations, while the horizontal bar chart displayed the prevalence of resistance for each antimicrobial agent. **(B)** Pie chart depicted the distribution of XDR *Salmonella* isolates from nine cities in Guizhou province. **(C)** Bar plot showed the detection rates of XDR *Salmonella* isolates from 2019 to 2023.

In total, 51 (5.5%; 51/931) *Salmonella* isolates exhibited XDR in Guizhou province, China, with being resistant to at least nine classes of antibiotics. All XDR *Salmonella* isolates showed 100% resistance to trimethoprim-sulfamethoxazole, streptomycin, tetracycline, ampicillin, and cefotaxime, and high resistance rates to chloramphenicol (98.0%), ampicillin/sulbactam (98.0%), azithromycin (99.2%), and ceftazidime (74.5%). Additionally, over 84% of isolates displayed resistance to ciprofloxacin and nalidixic acid. Notably, approximately 15.7% of isolates were resistant to colistin, while no resistance was observed for tigecycline, ceftazidime/avibactam, ertapenem, and meropenem (Figure 1A). Thirteen distinct antimicrobial resistance patterns were identified among these isolates, with the most prevalent pattern being C-SXT-CIP-NA-AZM-STS-TE-AM-CTX-CAZ-SAM (47.1%; 24/51) (Figure 1A and Supplementary Data S2). The analysis of XDR in clinical *Salmonella* isolates from nine cities in Guizhou between 2019 and 2023 revealed that the highest proportion of XDR isolates was in Zunyi at 35.3% (18/51), followed by Liupanshui (15.7%) and Guiyang (13.7%) (Figure 1B). Notably, the prevalence of XDR gradually increased from 3.8 to 7.4% between 2019 and 2023, with the exception of 2022 (Figure 1C).

### 3.2 Genomic characterization of XDR *Salmonella* isolates

A comprehensive WGS analysis was conducted on 51 XDR *Salmonella* isolates from Guizhou province. The basic genomic information and characteristics of these XDR *Salmonella* isolates were presented in Supplementary Data S3. Analysis of the genomic features revealed that the genomes of the 51 XDR *Salmonella* isolates ranged in size from 4.7 Mb to 5.4 Mb, with a guanine-cytosine (GC) content spanning from 51.71 to 52.30%. Additionally, the N50 values of these isolates fell within the range of 4,650,029 bp to 5,041,259 bp for the XDR isolates.

### 3.3 Serovar prediction, sequence type, and antimicrobial resistance pattern

**Figure 2:**
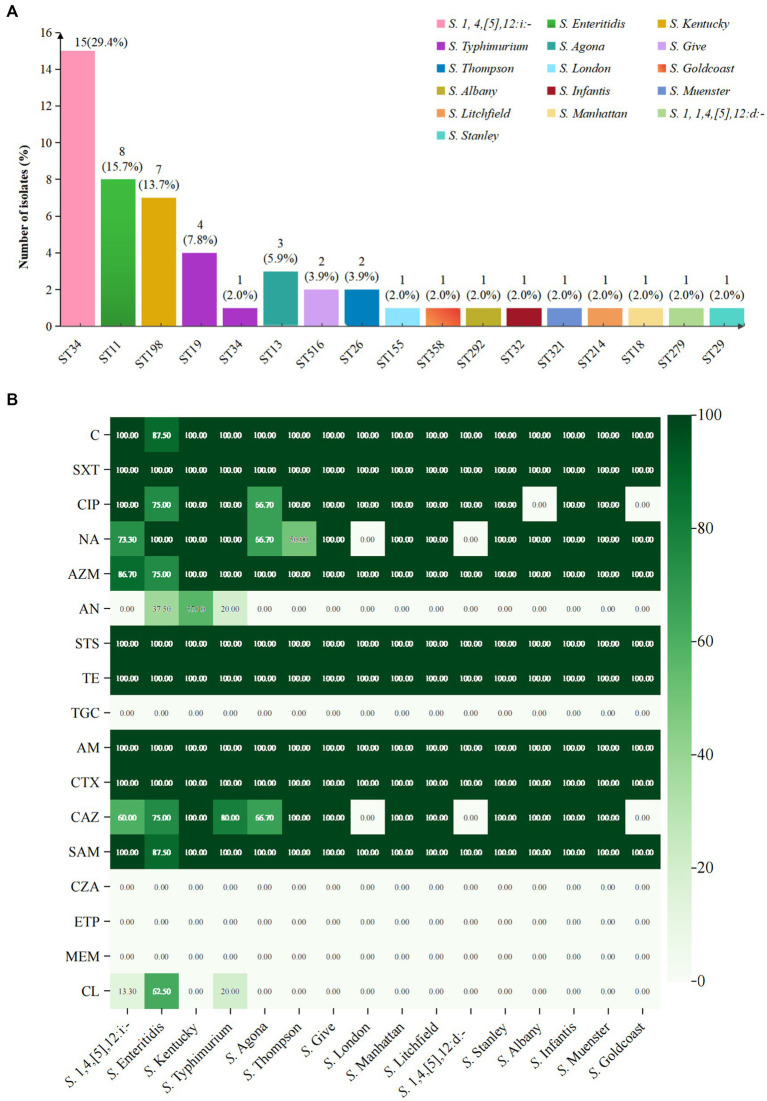
Analysis of serovars, STs, and antimicrobial resistance of 51 XDR *Salmonella* isolates. **(A)** Serovar prediction and MLST of 51 XDR *Salmonella* isolates. **(B)** The heatmap depicted the distribution of antimicrobial resistance across different serovars. The shades of color corresponded to the number of antimicrobial resistance isolates. Dark green indicated a high resistance rate, while white denoted the absence of resistance in that serovar.

Based on WGS data of 51 XDR *Salmonella*, 16 distinct serovars were identified. The most prevalent serovar was *S.* 1,4,[5],12:i:- (29.4%), followed by *S. Enteritidis* (15.7%) and *S.* Kentucky (13.7%) (Figure 2A). Moreover, the isolates were classified into 16 sequence types (STs), with ST34 (31.4%), ST11 (15.7%), and ST198 (13.7%) being the most common. Among the 16 distinct serovars, 15 serovars were linked to a specific ST type, except for *S. Typhimurium*, which included one ST34 isolate and four ST19 isolates (Figure 2A). Upon comparing antibiotic resistance phenotypes, it was observed that *S.* 1,4,[5],12:i:- isolates exhibited the broadest range of resistance rates, varying from 13.3 to 100.0%. Notably, colistin resistance was most pronounced in *S. Enteritidis* isolates, reaching 62.5%, while amikacin resistance was most prevalent in *S.* Kentucky at 57.1% (Figure 2B).

The analysis of antimicrobial resistance (AMR) patterns revealed distinct profiles among *Salmonella* serovars. *S.* 1,4,[5],12:i:- isolates displayed five unique AMR patterns, with the predominant pattern C-SXT-CTX-CAZ-TE-CIP-NA-AZM-STS-AM-SAM (53.3%) (Supplementary Data S2). In contrast to *S.* 1,4,[5],12:i:- isolates which demonstrated extensive multidrug resistance patterns, other serovars including *S.* Kentucky, *S. Typhimurium*, *S.* Agona, and *S.* Thompson exhibited comparatively fewer resistance profiles. All remaining analyzed serovars displayed a singular resistance pattern.

### 3.4 Associated antimicrobial resistance genes of XDR *Salmonella* isolates

#### 3.4.1 Distribution of antimicrobial resistance genes in 51 XDR *Salmonella* isolates

**Figure 3:**
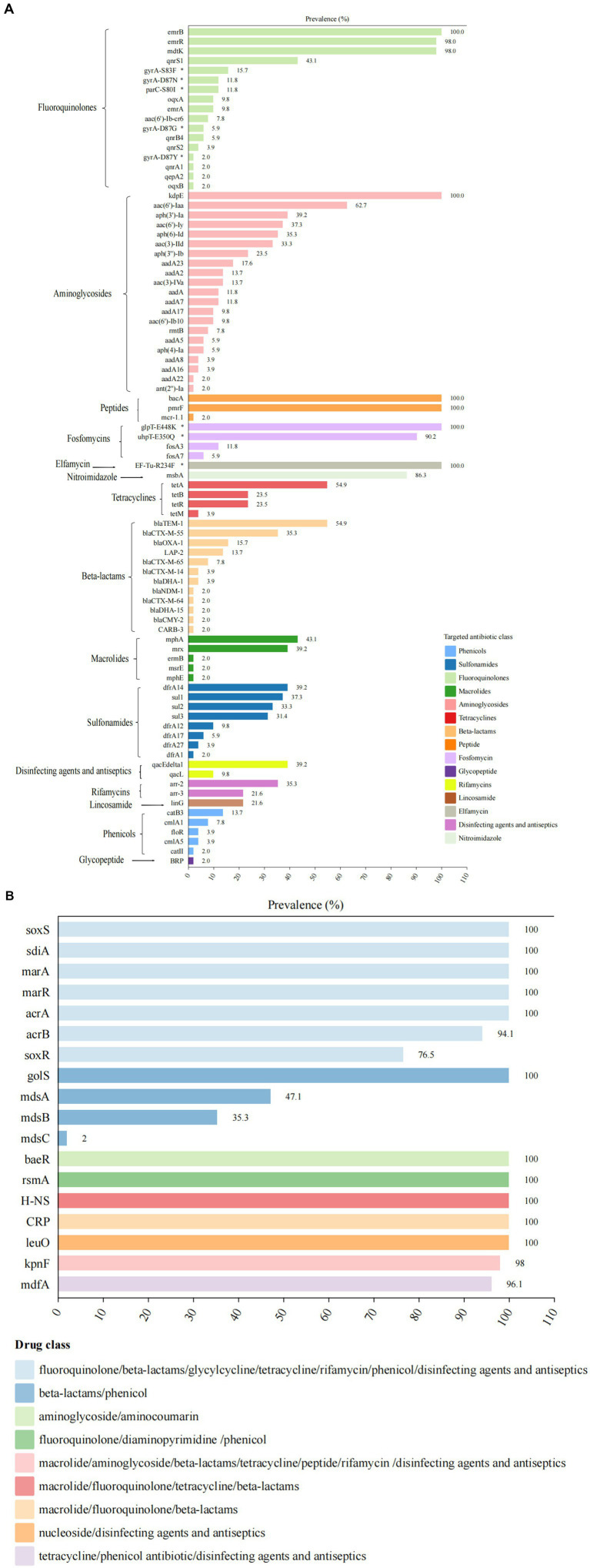
Antimicrobial resistance determinants among 51 XDR *Salmonella* isolates. **(A)** The distribution of ARGs and associated amino acid mutations, with corresponding genetic elements listed on the left vertical axis. The adjacent bar chart quantified their percentages, stratified by antimicrobial class through color-coded categorization. **(B)** Multidrug resistance genetic determinants present gene targets along the left margin. The accompanying visualization employs chromatic differentiation to demonstrate the proportional representation of MDR-associated genetic elements within the study.

Whole genome analysis of 51 XDR *Salmonella* isolates revealed 105 ARGs, including clinically significant point mutations in two chromosomal targets (*gyrA* and *parC*). These genetic determinants conferred resistance to 15 antimicrobial classes (Figure 3). Notably, 12 isolates harbored mutations in quinolone resistance-determining regions (*QRDRs*), with amino acid substitutions identified in both *gyrA* and *parC* subunits. These specific mutations were associated with reduced susceptibility to fluoroquinolones, a critically important class of antimicrobials in clinical practice. For *gyrA* gene, the identified amino acid substitutions were S83F (15.7%), D87N (11.8%), D87G (5.9%), and D87Y (2.0%). For *parC* gene, the S80I substitution was observed in 11.8% of isolates. Additionally, eight *PMQR* genes were identified, with the *qnrS* being the most prevalent (47.1%). The subtypes of the *qnrS* gene were *qnrS1* (43.1%) and *qnrS2* (3.9%). Other detected *PMQR* genes included *qnrB4* (5.9%), *qnrA1* (2.0%), *qepA2* (2.0%), *oqxA* (9.8%), and *oqxB* (2.0%), along with a variant of the aminoglycoside acetyltransferase gene *aac-(6′)-lb-cr* (7.8%). Moreover, various efflux pump resistance genes were detected in these isolates, such as *emrB* (100%), *emrR* (98%), and *mdtK* (98%). We found 21 ARGs associated with aminoglycoside resistance in 2.0 to 100% of these isolates. The *kdpE* gene was present in all isolates, while the *aac(6′)-Iaa* was present in 62.7%. For tetracycline resistance, related genes were identified in 86.3% (44/51) of the isolates. The *tetA* gene was present in over 54.9% of the isolates, while *tetB* and *tetR* were found in 23.5%, respectively. Regarding *β*-lactam resistance, 12 resistant genes were detected, with 78.4% (40/51) of the isolates carrying at least one *β*-lactam resistance gene. The most common was *bla_TEM-1_* (54.9%). Additionally, the *bla_CTX-M_* gene was detected in 49.0% of the isolates, with four subtypes identified: *bla_CTX-M-55_* (35.3%), *bla_CTX-M-65_* (7.8%), *bla_CTX-M-14_* (3.9%), and *bla_CTX-M-64_* (2.0%). The *bla_DHA_* gene was detected in 5.9% of the isolates, with subtypes of *bla_DHA-1_* (3.9%) and *bla_DHA-15_* (2.0%). The *bla_OXA-1_* gene was detected in 15.7% of the isolates, including *S.* 1,4,[5],12:i:-, *S. Typhimurium*, *S.* Kentucky, *S.* Thompson, and *S.* Stanley. We also identified the *bla_CMY-2_* gene in one *S*. Thompson isolate. Notably, the *bla_NDM-1_* gene was first detected in one *S. Typhimurium* isolate from Guizhou province. For macrolide resistance, the *mphA* and mrx genes are closely associated with azithromycin resistance, showing the detection rates at 43.1 and 39.2%, respectively. Both *mphA* and *mrx* genes were found to co-exist in 35.3% of the isolates. Additionally, we detected the *ermB*, *msrE*, and *mphE* genes, each at a rate of 2%. The most frequently identified sulfonamide resistance gene was *dfrA14*, which was present in 39.2% of the isolates. Additionally, the detection rates for the *sul1*, *sul2*, and *sul3* genes all exceeded 30%. Among the five phenicol genes examined, *catB3* was prevalent in 13.7% of the isolates, while the *cmlA* gene was detected in 11.8%. Furthermore, the subtypes of these resistance genes included *cmlA1* (7.8%) and *cmlA5* (3.9%). A gene that confers resistance to colistin, known as *mcr-1.1*, was identified in one *S.* 1,4,[5],12:i:- isolate. Besides, genes associated with resistance to fosfomycin, specifically *fosA3* and *fosA7*, were detected in 11.8 and 5.9% of isolates, respectively. Our study also revealed 18 multidrug resistance genes, with 11 of these genes found in all isolates (Figure 3B).

#### 3.4.2 The prevalence of ARGs in the top four common serovars

**Figure 4:**
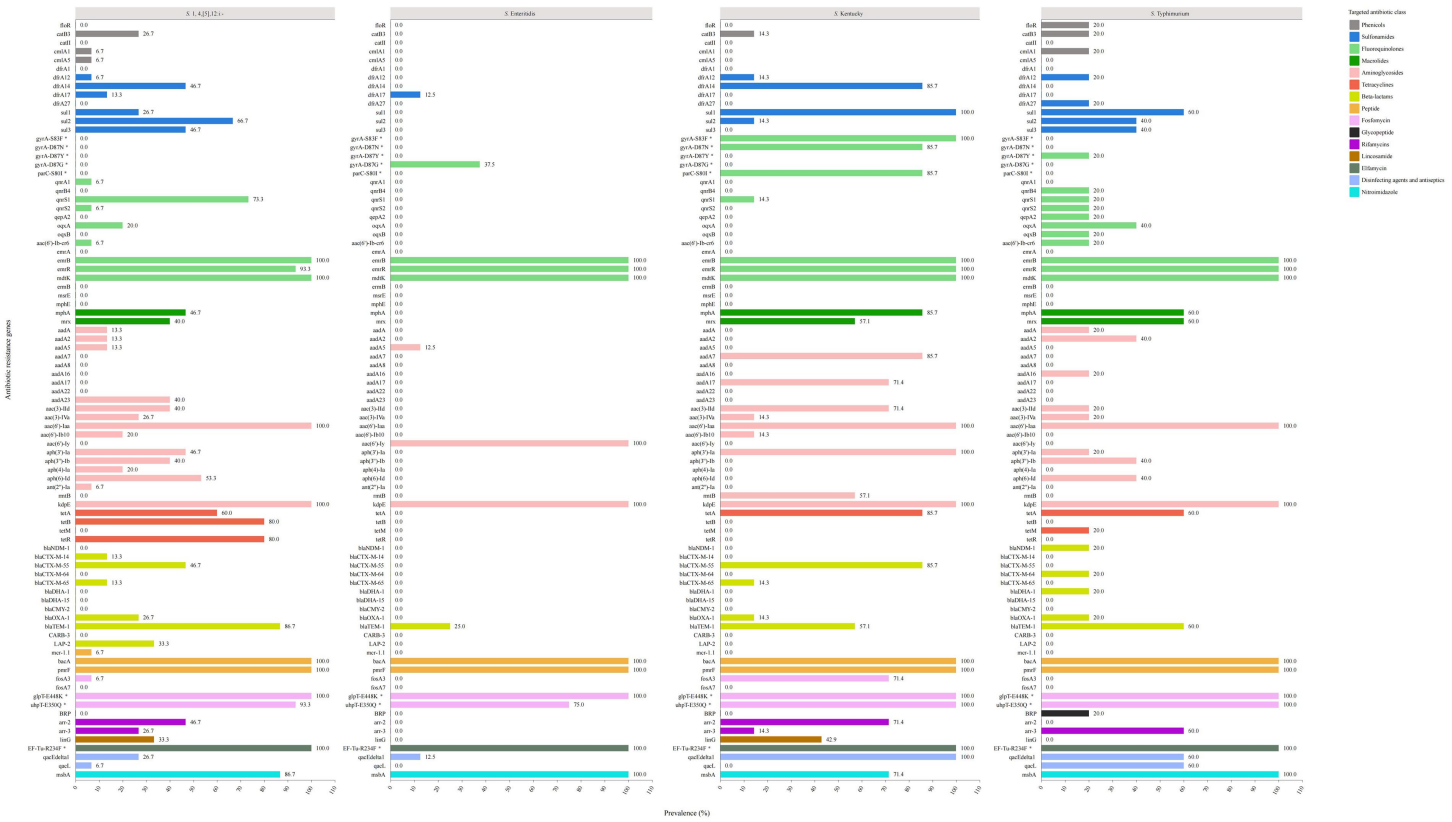
The prevalence of genetic determinants of antibiotic resistance in the top four common serovars of XDR *Salmonella*.

In our analysis, we compared the prevalence of ARGs among the four most common serovars of XDR *Salmonella* isolates (Figure 4), including *S.* 1,4,[5],12:i:-, *S. Enteritidis*, *S.* Kentucky, and *S. Typhimurium*. We observed significant polymorphisms in the ARGs across *S.* 1,4,[5],12:i:-, *S.* Kentucky, and *S. Typhimurium*. Regarding sulfonamide resistance, both *S.* 1,4,[5],12:i:- *and S. Typhimurium* harbored the *sul1*, s*ul2*, and *sul3* genes. The *sul2* gene was predominant in *S.* 1,4,[5],12:i:- (66.7%), while *sul1* was dominant gene in *S. Typhimurium* (60.0%). In contrast, *S. Enteritidis* exclusively possessed the sulfonamide resistance gene *dfrA17* and did not contain the *sul1, sul2,* or *sul3* genes. All *S.* Kentucky isolates carried the *sul1* gene, with some also carrying the *sul2* gene.

For fluoroquinolone (FQ) resistance, *S.* 1,4,[5],12:i:- exhibited five *PMQR* genes, with *qnrS1* (73.3%) being the predominant gene. Among *S. Enteritidis* isolates, 37.5% showed the *gyrA*-D87G point mutation without any additional *PMQR* genes, including *qnr*, *oqx*, and *aac(6′)-Ib-cr*. Notably, *S.* Kentucky isolates displayed three types of point mutations: 100% had *gyrA*-S83F, 85.7% had *gyrA*-D87N, and 85.7% had *parC*-S80I mutation. Furthermore, 14.3% of the *S.* Kentucky isolates carried the *qnrS1* gene. In contrast, *S. Typhimurium* isolates showed different patterns: 20.0% had the *gyrA*-D87Y point mutation, while another 20.0% carried a combination of *PMQR* genes, including *qnrB4*, *qnrS1*, *qnrS2*, *qepA2*, *oqxB*, and *aac(6′)-Ib-cr*. Additionally, 40.0% of the isolates carried the *oqxA* gene.

For macrolide resistance, *S.* 1,4,[5],12:i:-*, S.* Kentucky, and *S. Typhimurium* isolates contained the *mphA* and *mrx* genes, while none of the *S. Enteritidis* isolates carried these genes.

Regarding aminoglycoside resistance genes, *S.* 1,4,[5],12:i:- isolates harbored 14 genes. The most prevalent were *aac(6′)-Iaa* (100%) and *kdpE* (100%), followed by *aph(6)-Id* (53.3%), *aph(3′)-Ia* (46.7%), *aac(3)-IId* (40.0%), *aph(3′)-Ib* (40.0%), and *aadA23* (40.0%). In comparison, the *S. Enteritidis* isolates exhibited only three related genes. The *S.* Kentucky *and S. Typhimurium* isolates presented nine and ten genes, respectively. All *S.* Kentucky isolates carried the *aac(6′)-Iaa*, *kdpE*, and *aph(3′)-Ia* genes, while all *S. Typhimurium* isolates contained the *aac(6′)-Iaa* and *kdpE* genes.

For tetracycline resistance genes, *S.* 1,4,[5],12:i:- isolates showed a prevalence of 60.0% for the *tetA* gene and 80.0% for both the *tetB* and *tetR* genes. *S. Enteritidis* isolates did not possess any tetracycline resistance genes. Among *S.* Kentucky isolates, *tetA* was the predominant gene (85.7%), while *S. Typhimurium* isolates exhibited the *tetA* gene (60.0%) along with the *tetM* gene (20.0%).

Concerning *β*-lactam resistance genes, *S.* 1,4,[5],12:i:- isolates were found to harbor six different genes, with *bla_TEM-1_* being the most prevalent (86.7%), followed by *bla_CTX-M-55_* (46.7%). In contrast, *S. Enteritidis* isolates carried only *bla_TEM-1_* (25.0%). *S.* Kentucky isolates exhibited a diverse array of *β*-lactam resistance genes, including *bla_CTX-M-55_* (85.7%), *bla_TEM-1_* (57.1%), *bla_CTX-M-65_* (14.3%), and *bla_OXA-1_* (14.3%). For *S. Typhimurium* isolates, the most prevalent *β-*lactam resistance gene was *bla_TEM-1_* (60.0%), followed by *bla_CTX-M-64_* (20.0%), *bla_DHA-1_* (20.0%), *bla_OXA-1_* (25%), and *bla_NDM-1_* (20.0%).

### 3.5 Characterization of plasmids

In this study, 40 plasmids were identified in 51 XDR *Salmonella* isolates (Table 1). Of these isolates, 28 (54.9%) isolates contained at least one plasmid, while 23 (45.1%) had no plasmids. The 40 identified plasmids were classified into ten different categories: A (IncA), B (IncB/O/K/Z), C (IncC), H (IncHI2/IncHI2A), I (IncI1, IncI1-I), F (IncFII, IncFIB, IncFIA), Q (IncQ1), R (IncR), X (IncX1), and p0111. The most commonly found plasmid replicon was IncHI2/IncHI2A, present in 19 isolates (47.5%), followed by IncQ1 in six isolates (15.0%). Notably, one *S.* Goldcoast isolate carried a hybrid plasmid replicon, IncFIA(HI1)-HI1A-HI1B(R27), while one *S.* Litchfield isolate contained a hybrid plasmid replicon of type IncB/O/K/Z. Among the predominant serovar of the XDR *Salmonella* isolates, 15 *S.* 1,4,[5],12:i:- isolates exhibited the presence of IncHI2/IncHI2A (73.3%; 11/15), IncQ1 (33.3%; 5/15), and p0111 (6.7%; 1/15) plasmid replicons. Of the 40 plasmids identified, 20 were classified as conjugative, three as mobilizable, and the remaining as nontransferable. The most prevalent conjugative plasmid was IncHI2/IncHI2A (75.0%; 15/20), followed by IncI (15.0%; 3/20).

**Table 1:** Transfer mechanism of plasmids associated with XDR *Salmonella*.

Replicon types	Plasmid numbers	Antimicrobial resistance genes (ARGs)	Main ST types	Transfer mechanism	Conjugative transfer region			
					oriT	Relaxase	T4CP	T4SS
IncHI2/IncHI2A	19	*dfrA14 (11), dfrA12 (2), dfrA27, dfrA17 (2), sul3 (11), sul1 (8), sul2 (10), bla_CTX-M-65_ (3), bla_CTX-M-55_ (10), bla_TEM-1_ (7), bla_CTX-M-64_, bla_OXA-1_ (5), bla_NDM-1_, bla_CTX-M-14_ (2), qnrS2, qnrS1 (13), qnrA1, oqxA (4), oqxB, aac(6′)-Ib10 (5), aadA2 (2), aadA (3), aadA5 (2), aadA16, aadA3, aadA8, aadA22 (8), aph(3′)-Ia (13), aph(4)-Ia (3), aph(6)-Id (6), ant(2″)-Ia, aac(3)-IId (10), aac(3)-IVa (6), tetA (12), mphA (13), mrx (12), cmlA5, cmlA1 (2), catB3 (5), fosA3, BRP (MBL), mcr-1.1, arr-2 (11), arr-3 (7), linG (6), qacEdelta1 (8), qacL, LAP-2 (7)*	ST2 ST3	Conjugative (*n* = 15)	A/C	MOB_H_	*TrwB/TraD*	*Tra_F like*
				Non-Mobilizable (*n* = 4)	–	–	–	*Tra_F like*
IncFIA(HI1)/HI1A/HI1B(R27)	1	*dfrA12, sul3, sul2, bla_TEM-1_, qnrS1, aadA2, aadA, tetA, tetM, cmlA1, floR, qacL*	–	Conjugative	A/C	MOB_H_	*TrwB/TraD*	*Tra_F like*
IncF (IncFIB(S), *n* = 2), IncFII(S), *n* = 2, IncFIB(K), *n* = 1)	5	*dfrA27, sul1, sul2, bla_TEM-1_ (2), qnrS2, qnrB6, oqxA, aac(6′)-Ib10, aadA16, aac(3)-IId, aph(6)-Id, tetA, mphA, mrx, catII, arr-3, qacEdelta1*	–	Non-Mobilizable	–	–	*TraD* (*n* = 2)	*Tra_F like* (n = 4) *VirB like* (*n* = 1)
IncI1-I(Alpha), IncI(Gamma)	3	–	ST274	Conjugative	P-type	MOB_P_	*VirD4/TraG*	*Tra_I like*
IncX1	1	*dfrA14, sul3, bla_TEM-1_, tet(A)*	–	Mobilizable	NW-type	MOB_F_	*TrwB/TraD*	–
IncA/C	2	*dfrA12 (2), sul1 (2), sul2, bla_CMY-2_, bla_TEM-1_, bla_OXA-1_, bla_DHA-1_, qnrS1, qepA2, qnrB4, aac(6′)-Ib10, aadA2 (2), aph(3′)-Ia, aph(3″)-Ib (2), aph(6)-Id (2), tetA, mphA, mrx (2), catB3, arr-3, qacEdelta1*	ST11 ST3	Non-Mobilizable	–	–	–	–
IncB/O/K/Z	1	–	–	Conjugative	P-type	MOB_P_	*VirD4/TraG*	*Tra_I like*
IncR	1	*sul1, qnrB4, aph(3′)-Ia, tetA, mrx, arr-3*	–	Mobilizable	NW-type	MOB_F_	*TrwB/TraD*	–
p0111	1	*dfrA14, sul2, bla_TEM-1_, qnrS1, aph(3″)-Ib*	–	Mobilizable	A/C	MOB_H_	*TrwB/TraD*	–
IncQ1	6	–	–	Non-Mobilizable	–	MOB_H_ (*n* = 1)	*TrwB/TraD* (*n* = 1)	*Tra_F like* (*n* = 1)

### 3.6 The distribution of ARGs located on chromosomes and plasmids

**Figure 5:**
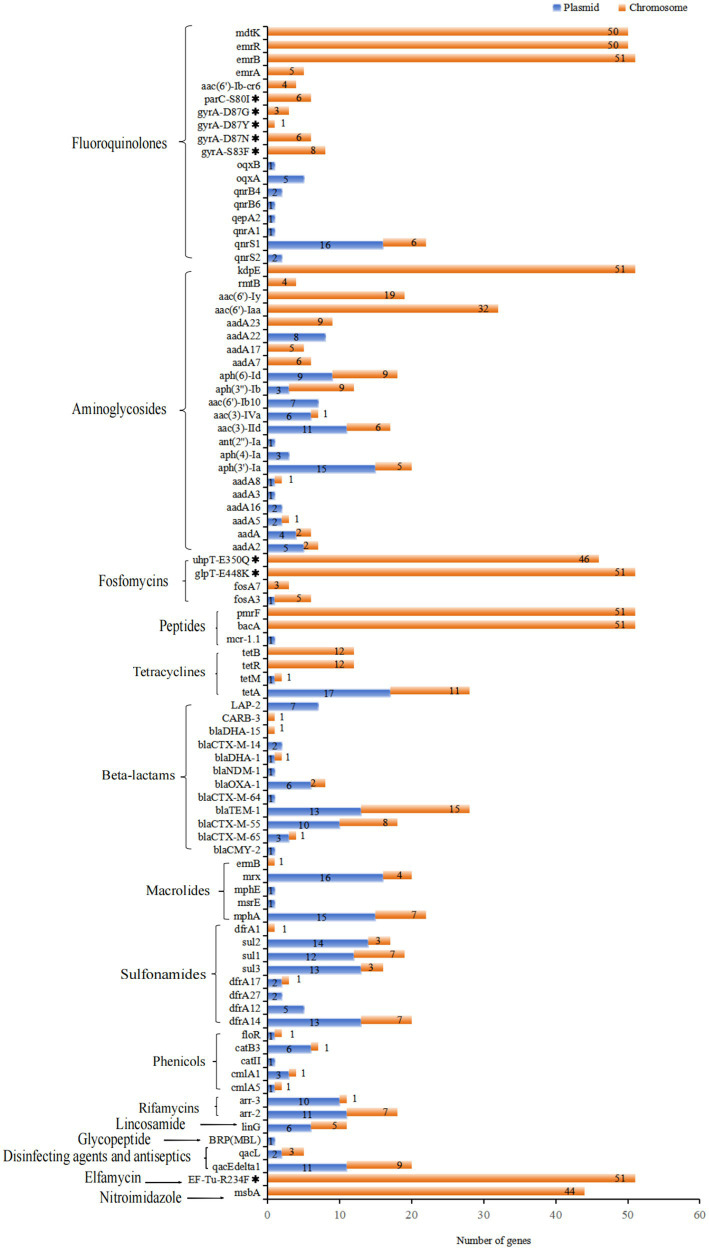
Distribution of ARGs and associated amino acid mutations located on chromosomes and plasmids of 51 XDR *Salmonella* isolates, targeting specific antimicrobial agents. Notably, 18 multidrug resistance genes were all located on chromosomal DNA, which did not show in this figure.

A total of 105 ARGs were identified in this study. Among these, 58 genes were located on plasmids, while 83 ARGs were found on chromosomes (Figure 5). In total, 45 ARGs were present in 20 conjugative plasmids. Of the 40 plasmids identified, 28 harbored at least one ARG, with a maximum of 20 genes present in a single plasmid (Supplementary Data S4). The plasmid genomes displayed an overrepresentation of ARGs, including *tetA*, *PMQR* genes, *aph(3′)-Ia*, *aadA2*, *mphA*, *mrx*, *sul1-3*, *dfrA14*, and *bla_TEM-1_*. Notably, several ARGs were found in both chromosomal and plasmid genomes, such as *bla_TEM-1_*, *tetA*, *aph (6)-Id*, *bla_CTX-M-55_*, and *mphA*. Among the 25 *bla_CTX-M_* positive isolates, *bla_CTX-M-55_* was present in 10 of these isolates, while *bla_CTX-M-65_* was found in three isolates, both located on IncHI2/IncHI2A plasmids. Additionally, 12 of these isolates contained conjugative plasmids. A significant finding was that specific ARGs were found in duplicate copies on distinct plasmids within the same isolate. For example, isolate SM2023030 exhibited two copies of the genes *sul1*, *mrx*, and *qacEdelta1*, whereas isolate SM2023155 had two copies of the gene *aph(3′)-Ia* on distinct plasmids. Moreover, the plasmid SM2021073-p1 carried the gene *mcr-1.1* along with 20 additional resistance genes, marking it the IncHI2/IncHI2A plasmid with the highest number of identified resistance genes in this study. Importantly, the resistance gene *bla_NDM-1_* located on an IncHI2/IncHI2A plasmid, was first detected in one *S. Typhimurium* isolate (SM2021168) from Guizhou province. This plasmid also carried 18 other resistance genes, including *oqxA/B*, *tetA*, *sul1-3*, *mphA*. Additionally, the IncFIA(HI1)-HI1A-HI1B(R27) plasmid contained both the *tetA* and *tetM* genes, which was the only plasmid in this study to carry two tetracycline resistance genes. It was also noteworthy that one *S.* Thompson isolate, SM2023030, identified the *bla_CMY-2_* gene on its IncC plasmid.

### 3.7 CgMLST cluster analysis of XDR *Salmonella* in Guizhou

**Figure 6:**
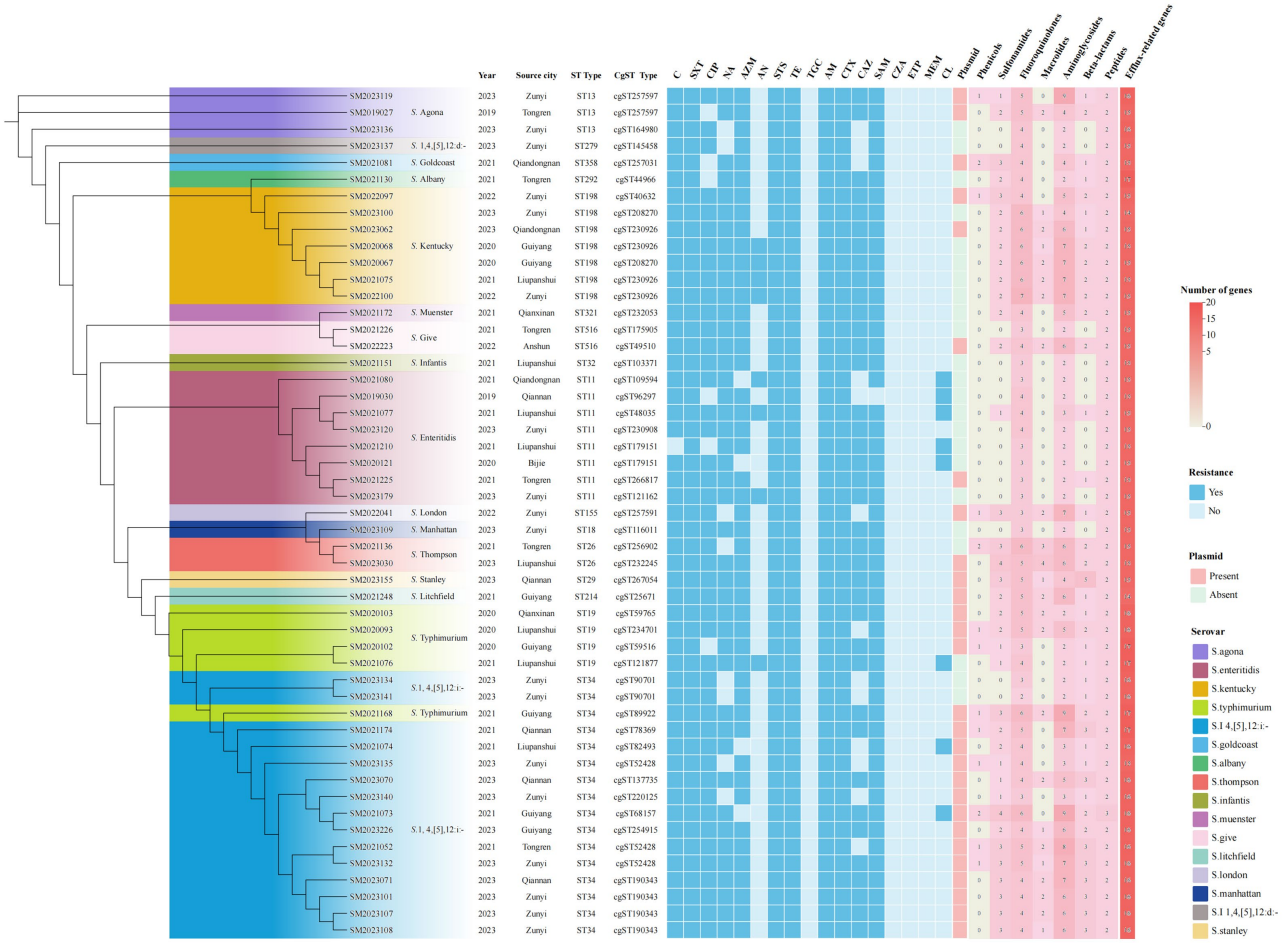
Phylogenetic analysis of 51 XDR *Salmonella* isolates using core genome MLST. The ML tree was constructed based on cgMLST profiles, with branch colors representing distinct serovars. Antimicrobial resistance phenotypes were depicted using square markers: filled blue squares indicated positive resistance phenotypes, while unfilled blue squares indicated susceptible isolates. The rightmost panel illustrated plasmid carriage and resistance gene distribution, where pink squares represented concurrent presence of both plasmids and resistance genes. Numeral within squares indicated the quantity of resistance genes corresponding to specific antibiotic classes.

The cgMLST clustering tree was constructed based on differences in the core genomes of the isolates, as well as an analysis of their background information, antimicrobial resistance patterns, and the classification of associated resistance genes in the 51 XDR isolates (Figure 6). The cgMLST analysis revealed that 51 XDR *Salmonella* isolates from Guizhou were divided into 39 cgSTs. Among these, seven cgSTs contained more than two isolates. Cluster analysis of all isolates based on cgSTs indicated that isolates of the same serovar were likely homologous. Notably, the *S.* 1,4,[5],12:i:- and *S. Typhimurium* isolates exhibited similar genomic relatedness on the phylogenetic tree. Isolates of *S.* Stanley, *S.* Litchfield, *S. Typhimurium*, and *S.* 1,4,[5],12:i:- were clustered together in the same clade. Sixteen ST34 isolates were divided into 10 cgSTs, with the dominant type being cgST190343 (25%, 4/16). Additionally, three cgSTs contained more than two isolates. Four cgST190343 isolates, SM2023071, SM2023101, SM2023107, and SM2023108, showed identical resistance profiles and carried the IncHI2/IncHI2A plasmids. These IncHI2/IncHI2A plasmids were found to possess genes such as *dfrA14*, *sul3*, *sul2*, *bla_CTX-M-55_*, *bla_TEM-1_*, *qnrS1*, *aadA22*, *aph(3′)-Ia*, *aac(3)-IId*, *aph(6)-Id*, *tetA*, *mphA*, *arr2*, *linG*, and *LAP2 genes*, suggesting that these isolates may have originated from the same source of infection. Among the four cgST190343 isolates, geographical distribution analysis revealed one isolate originated from Qiannan Autonomous prefecture while three were clustered in Zunyi city, indicating possible inter-regional dissemination of this isolate. Furthermore, the two cgST90701 *S.* 1,4,[5],12:i:- isolates, SM2023134 and SM2023141, exhibited identical antimicrobial resistance profiles and harbored concordant resistance determinants, implying a shared infection origin.

## 4 Discussion

A tremendous increase in antimicrobial resistance among *Salmonella* isolates poses a significant global concern (Algammal et al., 2023; Chen et al., 2022; Cummins et al., 2020; Karampatakis, 2024; Liu B. et al., 2024; Sallam et al., 2024). In Guizhou province, surveillance data revealed a concerning escalation in XDR *Salmonella* prevalence, with detection rates climbing from 4.4% during 2013–2018 to 5.5% in the subsequent 2019–2023 period (Wei et al., 2023). Notably, our study revealed an accelerated upward trajectory, showing the detection rate of XDR *Salmonella* isolates nearly doubling from 3.8% in 2019 to 7.4% by 2023 within the same province. Due to their resistance to multiple antibiotic agents, the effective treatment of *Salmonella* infections was threatened, thus increasing the morbidity and mortality of patients. However, in 2022, the number of XDR isolates decreased, which may be attributed to environmental hygiene measures, less eating out, less travel, and less turnover during the pandemic of COVID-19. These changes highlighted the critical role of human mobility in the spread of resistant pathogens. In this study, we focused on XDR *Salmonella* isolates from humans because of the lack of data on this particular *Salmonella* phenotypes and resistance genes in Guizhou province and due to its severity in cases of outbreaks and surveillance. Limited data are available on genomic resistance characterization. Therefore, we investigated 51 XDR human *Salmonella* isolates from Guizhou province between 2019 and 2023 regarding genomic characterization, phylogenetic relationships, and resistance genes on chromosomes and plasmids. Understanding the underlying drug resistance mechanisms of these pathogens helps us finding solutions for the long-standing antibiotic resistance problem. This is the first study on genomic epidemiology of XDR human *Salmonella* isolates in Guizhou province, China.

In the present study, we noted that the prevalence of XDR *Salmonella* varied significantly across different regions, with the highest proportion of XDR isolates observed in Zunyi city, reaching up to 35.3%. These variations might be attributed to a combination of factors, including the distinctive geographical characteristics, the diverse habits of food consumption among local populations, as well as the climatic conditions. The climatic conditions in each region, including temperature, humidity, and seasonal changes, can greatly affect the survival and proliferation of XDR *Salmonella*. Zunyi city, located in the northern region of Guizhou province, is warmer and more humid conditions, which may contribute to a longer survival period for pathogens outside their host, potentially accelerating their transmission rates. Serological identification of 51 XDR *Salmonella* isolates revealed that *S.* 1,4,[5],12:i:-, *S. Enteritidis*, and *S.* Kentucky were the most frequent serovars, with ST34, ST11, and ST198 being the predominant sequence type, which was consistent with previous reports in Guangdong, Jiangsu, and other European countries, indicating a widespread clonal (Sun et al., 2024; Liu B. et al., 2024; European Food Safety Authority (EFSA) and European Centre for Disease Prevention and Control (ECDC), 2019). These findings highlight the critical role of regional-specific environmental and behavioral factors in shaping the distribution and prevalence of XDR *Salmonella.*

The WGS analysis of 51 XDR *Salmonella* isolates revealed an extensive AMR landscape, with 105 ARGs conferring resistance to 15 antimicrobial classes. When examining genetic determinants contributing to resistance against FQs, we noted the presence of double mutations in the *gyrA* (D87 and S83) and one mutation in *parC* (S80I) in XDR isolates. These mutations were regarded as significant target-site alterations, which was consistent with findings from previous studies (Abd El-Aziz et al., 2021; Chen et al., 2024; Petrin et al., 2023). Furthermore, the *PMQR* genes determinants lead to decreased susceptibility to FQs and facilitate the selection of higher levels of FQ resistance (Chang et al., 2021; Vázquez et al., 2022). In the current study, eight *PMQR* genes were detected, with *qnrS* being the most prevalent in 47.1% of the isolates. Additionally, *qnrS1* was identified among the examined isolates from various serovars, consistent with previous studies (Chen K. et al., 2020; Chen Z. et al. 2020). Meanwhile, it was important to note that *qnr* genes were found on multiple plasmids, which can disseminate across different regions and sources (She et al., 2023). In our study, 75.0% of the identified conjugative plasmid types were IncHI2/IncHI2A. Previous studies indicated that the IncHI2/IncHI2A plasmid was an essential lineage contributing to the spread of antibiotic resistance in *Salmonella*, suggesting that *qnr* genes could undergo horizontal gene transfer through conjugation between bacteria, thus promoting the spread of resistance genes (She et al., 2023).

In our study, the *bla_TEM-1_* gene conferring ampicillin resistance was the most common *β*-lactam resistance gene, consistent with its global dominance in *Salmonella* serovars (Chen et al., 2024). Meanwhile, we identified four subtypes of *bla_CTX-M_*, a critical resistance gene family associated with third-generation cephalosporins resistance. Among these, *bla_CTX-M-55_* gene was predominant, mirroring its high prevalence in *Salmonella* isolates from Australia and in zoonotic and human isolates in China (Ingle et al., 2021; Sun et al., 2024; Zhou et al., 2023). The selective pressure from extensive clinical and veterinary use of third-generation cephalosporins has driven the global rise of *CTX-M* extended-spectrum *β*-lactamases (ESBLs), displaying traditional resistance determinants like *TEM* and *SHV* variants as the dominant ESBLs (Yu et al., 2024). This epidemiological shift was further exacerbated by the genetic mobility of *bla_CTX-M_* genes, which were frequently plasmids-mediated and capable of horizontal tranfer between bacterial isolates through conjugation (Wei et al., 2024; Yu et al., 2024). The increasing prevalence of *bla_CTX-M_* variants among *Salmonella* isolates poses a growing public health crisis, as these multidrug-resistant pathogens threatened the efficacy of frontline *β*-lactam therapies worldwide.

The production of AmpC *β*-lactamases has become a critical resistance mechanism in Gram-negative bacilli, conferring broad-spectrum resistance to *β*-lactam antibiotics. Of particular concern is the plasmid-mediated *bla_CMY-2_* gene, which encoded an AmpC-type enzyme capable of hydrolyzing extended-spectrum cephalosporins. While *bla_CMY-2_* has been extensively documented in food-producing animals and retail meat products across China, South Korea, and Spain (Na et al., 2020; Vázquez et al., 2021; Zhang et al., 2024b), its detection in human clinical isolates remains uncommon. Recent evidence from China, however, has confirmed the emergence of *bla_CMY-2_*-harboring *Salmonella* in human infections (Zhang et al., 2024a), highlighting potential zoonotic transmission pathways. In this study, we identified one clinically derived *S*. Thompson isolate carrying *bla_CMY-2_* on a IncC-type plasmid. Notably, this isolate concurrently harbored a distinct plasmid bearing macrolide resistance determinants (*mphA*, *msrE*, *mphE*, and *mrx*), creating a multidrug-resistant genetic configuration. The dual-plasmid system provided compelling evidence for horizontal gene transfer between environmental and clinical reservoirs. The colocalization of *β*-lactamase and macrolide resistance genes on mobile genetic elements raised critical concerns about coselection pressures that may perpetuate resistance traits, even under targeted antimicrobial stewardship. These findings highlighted the urgent priorities that enhanced surveillance of plasmid-mediated AmpC dissemination across the food-animal-human interface and implementation of stricter antimicrobial usage policies to disrupt resistance transmission networks.

Additionally, we identified one *S. Typhimurium* isolate carrying 18 ARGs, including *bla_NDM-1_*, *oqxA/B*, and *mphA*, on the IncHI2/IncHI2A plasmid for the first time. This isolate exhibited high resistance to third-generation cephalosporins (ceftazidime and cefotaxime), ciprofloxacin, azithromycin, and eleven other antimicrobials. The co-existence of ESBLs genes with other clinically significant ARGs, including *qnr* genes, was observed in recent studies (Li et al., 2022; Luo et al., 2020). The *bla_NDM-1_* gene confers resistance to many *β*-lactam antimicrobials, including carbapenems (Pitout, 2012). The production of *NDM* by *Salmonella* spp. has been reported in the United States, China, and Singapore (Rahman et al., 2024; Song et al., 2023; Octavia et al., 2020). Infections caused by *bla_NDM-1_* producers are difficult to treat, leading to increased morbidity, prolonged hospital stays, higher medical costs, and mortality due to ineffective standard treatments, which significantly affect treatment options and clinical outcomes (Octavia et al., 2020; Rahman et al., 2024; Song et al., 2023). The detection of such multidrug-resistant configurations in human pathogens emphasizes the need for One Health approaches to combat antimicrobial resistance.

The major determinants of AMR in the XDR isolates studied included genes associated with resistance to aminoglycosides, tetracyclines, and sulfonamides. Most of these genes have been documented in previous research. For example, the chromosomally encoded gene *aac(6′)-Iaa* was identified in *S.* 1,4,[5],12: i: - (Wei et al., 2024) and *S. Typhimurium* (Akinyemi et al., 2023) from human origin across multiple countries. Phenotypic resistance to tetracycline correlated with known genetic determinants, particularly the efflux pump gene *tetA,* which dominated observed resistance patterns (Zhao et al., 2022), a finding consistent with our data. While sulfonamide resistance was primarily associated with the genes *sul1* and *sul2* (Chen Z. et al., 2020), our study identified *dfrA*14 as the most prevalent sulfonamide resistance gene, contrasting with the expected predominance of *sul1*, *sul2*, and *sul3*. Notably, the macrolide resistance gene *mphA* was detected in 43.1% of the XDR *Salmonella* isolates. Its widespread presence raised clinical concerns, as *mphA* threatened the effectiveness of azithromycin for treating *Salmonella* infections (Wang et al., 2023). However, the mechanisms driving *mphA* dissemination remain unclear, highlighting the need for more in-depth comprehensive molecular epidemiological studies to elucidate transmission pathways. Both *fosA3* and *fosA7* fosfomycin resistance genes were identified, posing theoretical challenges for managing XDR *Salmonella* infections. Due to the absence of a fosfomycin-resistant phenotype in our isolates, we can not deterninate the clinical relevance of this fosfomycin resistance genes. These findings highlighted the importance of antimicrobial susceptibility testing when whole-genome sequencing might not provide a definitive assessment (Wang J. et al., 2024). Of particular concern was the detection of the plasmid-mediated *mcr-1.1* colistin resistance gene in one *S.* 1,4,[5],12:i:- isolate. As colistin remains a last-resort antibiotic, this finding signals an alarming escalation in resistance to critically important antimicrobials (Liu J. H. et al., 2024).

The analysis of the four prevalent serovars harboring ARGs revealed that *S*. 1,4,[5],12:i:-isolates exhibited a broader range of resistance determinants compared to other serovars. In contrast, *S. Enteritidis* isolates contained fewer ARGs. Interestingly, when we analyzed the genetic factors contributing to FQs resistance, isolates from *S.* 1,4,[5],12:i:- and *S. Typhimurium* were primarily characterized by the presence of *qnr* genes. On the other hand, 87.5% of *S.* Kentucky isolates displayed double mutations in the *gyrA* gene (D87N and S83F) and a single mutation in the *parC* (S80I) gene. These mutations could be significant genetic factors contributing to FQs resistance, which was consistent to the dominant clone of *S.* Kentucky ST198 in Europe (European Food Safety Authority (EFSA) and European Centre for Disease Prevention and Control (ECDC), 2019). Our findings revealed that the diversity of serovars was associated with different resistance genes and mechanisms. Notably, both *tetB* and *tetR* genes coexisted in *S.* 1,4,[5],12:i:-. Previous studies identified *tetB* in clinically significant clones of *S. Typhimurium* and its monophasic variant (Petrin et al., 2023). This suggested that there was genetic heterogeneity among the different serovars, likely a result of their adaptation and evolution in response to varying environmental and antimicrobial pressures. This heterogeneity was reflected in the distinct distribution and transmission patterns of drug resistance genes, which might be facilitated by horizontal gene transfer between isolates.

The dissemination of AMR was, in part, attribuable to the horizontal transfer of ARGs, frequently facilitated by plasmids (Castañeda-Barba et al., 2024). We conducted a further analysis of the chromosomal and plasmid-borne distribution of ARGs in 51 XDR *Salmonella* isolates from Guizhou province using WGS data. Our findings revealed that over half of the isolates harbored at least one plasmid, with IncHI2/IncHI2A plasmids being particularly prevalent, which indicated their widespread distribution among XDR human *Salmonella* isolates in this region. Notably, the carriage rate of IncHI2/IncHI2A plasmids in China significantly exceeded those reported in other countries (Sun et al., 2024). These plasmids were known to facilitate interbacterial ARG transfer through conjugation, accelerating the dissemination of resistance and posing a serious public health threat (Jiang et al., 2020). Several crucial ARGs identified on IncHI2/IncHI2A plasmids in Guizhou included *tetA*, *PMQR* genes, *bla_CTX-M_*, *aph(3′)-Ia*, *mphA*, *sul1-3*, *dfrA14*, *bla_TEM-1_*, *mcr-1.1*, and *bla_NDM-1_*. The accumulation of such ARGs on these plasmids enabled bacterial resistance to first-line antimicrobial, complicating clinial treatment (Wang and Dagan, 2024). Of particular concern was the detection of IncFIA(HI1)-HI1A-HI1B(R27) hybrid plasmid, which have been linked to the spread of high-risk ARGs like tet (X4) in *Enterobacterales* from both human and nonhuman sources (Yue et al., 2024; Zhai et al., 2024). These plasmids were classified as high-risk due to their association with clinically significant resistance mechanisms. These findings highlight the urgent need for sustained surveillence of plasmid-mediated ARGs to identify emerging resistance trends and inform targeted interventions.

Molecular typing for *Salmonella* isolates within the same serovars is critical for outbreak investigation and bacterial epidemiology. With the widespread application of WGS, WGS-based high-resolution subtyping methods have become popularized in tracing bacterial transmission and identifying outbreaks. Among these methods, cgMLST is the most widely used approach for the strain-level differentiation of *Salmonella*. This method offers high discriminatory power, making it invaluable in epidemiological and outbreak investigations (Zhao et al., 2022). CgMLST is analyzed variations in core genome loci (defined as genes present in 95–98% of the reference isolates used to establish the allele scheme) to construct phylogenetic relationships (Dangel et al., 2019). In this study, cgMLST analysis of 51 XDR *Salmonella* isolates revealed significant genetic diversity, with 39 distinct cgSTs identified. No evidence of localized outbreaks was observed in Guizhou. Four isolates of cgST190343 exhibited identical resistance profiles and carried IncHI2/IncHI2A plasmids with the same ARGs patterns. These isolates were originated from two distinct cities in 2023, indicating either the same source of infection or potential cross-regional transmission. The cgMLST results demonstrated robust discriminatory resolution, underscoring its utility in public health surveillance. By contributing genomic data from these XDR *Salmonella* isolates to global databases, we can enhance resources for pathogen tracking and strength international disease surveillance systems. Such efforts enable faster, more targeted public health interventions during outbreaks and improve preparedness for emerging antimicrobial resistance pathogens.

## 5 Conclusion

Our study presents the first data on the WGS-based characterization of XDR *Salmonella* isolates from humans between 2019 and 2023 in Guizhou province, China. The detection rate of XDR *Salmonella* isolates increased from 3.8% in 2019 to 7.4% in 2023. *S*. 1,4,[5],12:i:- isolates exhibited a broader range of resistance determinants than other serovars. Notably, one *bla_NDM-1_* positive isolate was detected for the first time in Guizhou. Meanwhile, XDR *Salmonella* isolates carried multiple ARGs on plasmids, which could promote the acquisition and dissemination of ARGs. This study showed that implementing continuous dynamic surveillance will enhance our understanding of the epidemiology of XDR *Salmonella* in this region and help public health officials identify emerging trends and potential threats.

## Data Availability

The original contributions presented in this study were included in the article and its Supplementary material. The sequence data presented in this study were publicly available in figshare: https://doi.org/10.6084/m9.figshare.28521008. Further inquiries can contact the corresponding author.

## Footnotes

^1^ https://whonet.org/software.html

^2^ https://github.com/DerrickWood/kraken2

^3^ https://github.com/fenderglass/Flye

^4^ https://github.com/nanoporetech/medaka

^5^ http://www.denglab.info/SeqSero2

^6^ https://cge.food.dtu.dk/services/MLST/

^7^ https://card.mcmaster.ca/

^8^ https://github.com/smaegol/PlasFlow

^9^ https://cge.food.dtu.dk/services/PlasmidFinder/

^10^ https://bioinfo-mml.sjtu.edu.cn/oriTDB/index.php

^11^ https://bioinfo-mml.sjtu.edu.cn/SecReT4/

^12^ https://bitbucket.org/genomicepidemiology/cgmlstfinder

^13^ http://www.iqtree.org

^14^https://www.chiplot.online/tvbot.html.
